# Is hematopoietic stem cell transplantation a therapeutic option for mucolipidosis type II?

**DOI:** 10.1016/j.ymgmr.2020.100704

**Published:** 2021-01-14

**Authors:** Luise Sophie Ammer, Sandra Pohl, Sandra Rafaela Breyer, Charlotte Aries, Jonas Denecke, Anna Perez, Martin Petzoldt, Johanna Schrum, Ingo Müller, Nicole Maria Muschol

**Affiliations:** a Department of Pediatrics, University Medical Center Hamburg-Eppendorf, Hamburg, Germany; b International Center for Lysosomal Disorders (ICLD), University Medical Center Hamburg-Eppendorf, Hamburg, Germany; c Department of Osteology and Biomechanics, University Medical Center Hamburg-Eppendorf, Hamburg, Germany; d Department of Pediatric Orthopedics, Children's Hospital Altona, Hamburg, Germany; e Department of Orthopedics, University Medical Center Hamburg-Eppendorf, Hamburg, Germany; f Department of Anesthesiology, University Medical Center Hamburg-Eppendorf, Hamburg, Germany; g Division of Pediatric Stem Cell Transplantation and Immunology, University Medical Center Hamburg-Eppendorf, Hamburg, Germany

**Keywords:** Mucolipidosis type II, I-cell disease, Lysosomal storage disorder, Hematopoietic stem cell transplantation, Bone marrow cell transplantation, Treatment, Cognitive function, Life quality

## Abstract

**Background:**

Mucolipidosis type II (MLII) is an ultra-rare lysosomal storage disorder caused by defective lysosomal enzyme trafficking. Clinical hallmarks are craniofacial dysmorphia, cardiorespiratory dysfunction, hepatosplenomegaly, skeletal deformities and neurocognitive retardation. Death usually occurs in the first decade of life and no cure exists. Hematopoietic stem cell transplantation (HSCT) has been performed in few MLII patients, but comprehensive follow-up data are extremely scarce.

**Methods:**

MLII diagnosis was confirmed in a female three-month-old patient with the mutations c.2213C > A and c.2220_2221dup in the *GNPTAB* gene. At nine months of age, the patient received HSCT from a 9/10 human leukocyte antigen (HLA)-matched unrelated donor.

**Results:**

HSCT resulted in a sustained reduction of lysosomal storage und bone metabolism markers. At six years of age, the patient showed normal cardiac function, partial respiratory insufficiency and moderate hepatomegaly, whereas skeletal manifestations had progressed. However, the patient could walk and maintained an overall good quality of life. Neurocognitive testing revealed a developmental quotient of 36%. The patient died at 6.6 years of age following a human metapneumovirus (hMPV) pneumonia.

**Conclusions:**

The exact benefit remains unclear as current literature vastly lacks comparable data on MLII natural history patients. In order to evaluate experimental therapies, in-depth prospective studies and registries of untreated MLII patients are indispensable.

## Graphical abstract

**Figure:**
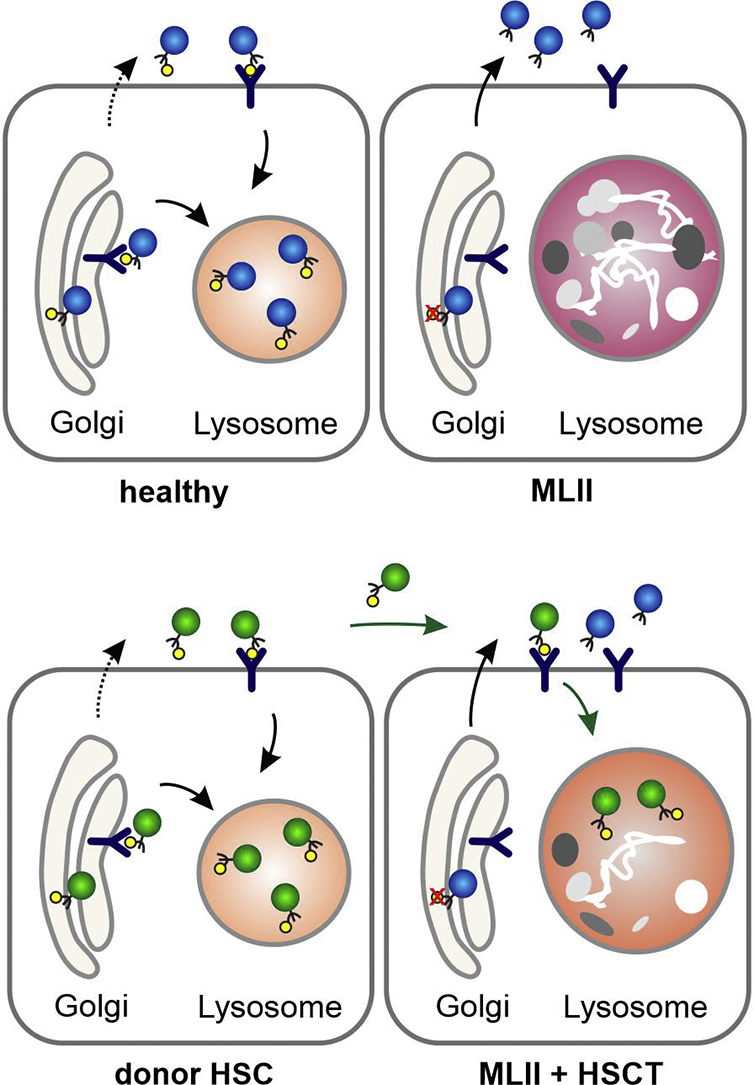

## 1 Introduction

**Fig. 1:**
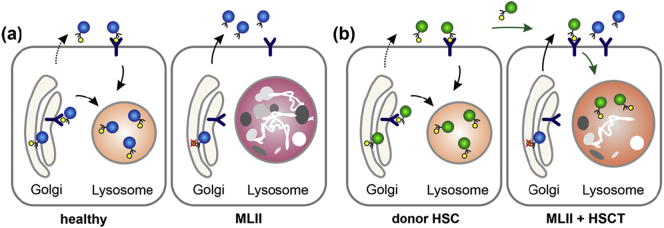
Targeting of lysosomal enzymes in health and disease (**a**) GlcNAc-1-phosphotransferase is involved in tagging mannose 6-phosphate (M6P) residues (yellow circles) on lysosomal enzymes (blue circles) for their targeting to lysosomes by M6P-receptors. In MLII, missorting of multiple lysosomal enzymes lacking M6P-residues due to reduced GlcNAc-1-phosphotransferase activity results in accumulation of macromolecules in lysosomes. (**b**) Hematopoietic donor cells produce functional M6P-containing lysosomal enzymes (green circles), which are taken up by MLII cells and transported to the lysosomes for degradation of macromolecules. Of note, hypersecretion of M6P-lacking lysosomal enzymes is not prevented despite metabolic cross-correction.

Mucolipidosis type II (MLII, I-cell disease, MIM# 252500), a rare autosomal recessive lysosomal storage disorder, is caused by mutations in the *GNPTAB* gene, which encodes the α/β-subunit precursor of the *N*-acetylglucosamine (GlcNAc)-1-phosphotransferase. This enzyme catalyzes the first step in the generation of mannose 6-phosphate (M6P) residues on newly synthesized lysosomal enzymes for their efficient targeting to lysosomes (Fig. 1A) [1]. In MLII patients, the missorting of multiple lysosomal enzymes results in accumulation of non-degraded macromolecules in lysosomes impairing cellular function (Fig. 1A). Clinically, MLII manifests as a progressive multi-systemic disease with prenatal or neonatal onset. Hallmarks are facial dysmorphia with prominent gingival hyperplasia, cardiorespiratory dysfunction, hepatosplenomegaly, severe skeletal pathologies and a global developmental delay. Major skeletal abnormalities comprise growth impairment, thoracic and long bone deformity, a variety of hip pathologies and progressive joint contractures [[1], [2], [3], [4]]. Death usually occurs in early childhood due to cardiopulmonary complications [1]. To date, no therapy exists for MLII, not even in the context of clinical trials.

Hematopoietic stem cell transplantation (HSCT) has been performed for over 30 years in various lysosomal storage disorders [5]. In children with mucopolysaccharidosis type IH (MPSIH, Hurler syndrome) below 2.5 years of age, HSCT has been established as the treatment of choice, mainly, because it halts progressive neurodegeneration [6]. Hematopoietic donor cells produce functional M6P-containing lysosomal enzymes, which are taken up intracellularly and transported to the lysosomes for degradation of macromolecules (Fig. 1B) [7]. Hereby, metabolic “cross-correction” is achieved. Although the efficacy of HSCT has been explored in MLII [[8], [9], [10], [11], [12], [13]], in-depth data are still scarce. This report summarizes the outcome of a 6.3-year-old MLII patient, who received HSCT at 9 months of age with the intention to preserve neurocognitive function.

## 2 Materials and methods

### 2.1 Patient and diagnosis

The female patient is the first child of non-consanguineous caucasian parents and was born spontaneously at term. The newborn presented with a birth weight of 3000 g (25th percentile), a body height of 46 cm (<1st percentile) and an occipitofrontal circumference (OFC) of 32 cm (<1st percentile). Prenatal ultrasound had already indicated skeletal abnormalities. The postnatal physical examination confirmed micromelia, thoracic deformity, femoral bowing and joint contractures. Facial dysmorphia comprised coarsenings, periorbital padding and gingival hyperplasia. Plain radiography revealed dysostosis multiplex*.* Standard biochemical testing [14] of lysosomal enzyme activities in the patient's serum proved their massive hypersecretion (Fig. 1A, Table 1). Sanger sequencing of the *GNPTAB* gene at six weeks of age identified the compound heterozygous mutations c.2213C > A and c.2220_2221dup, which were previously found in Chinese and Argentinian patients with MLII [1,15,16]. The loss-of-function mutations on both alleles (Fig. S1) predicted the manifestation of a severe phenotype.

**Table 1:** Serial biochemical results.

	**Age**	**GAGs/crea**	**Dpd/crea**	***α-Idu***	**IDS**	**ASA**	**ß-Gal**
	month(s)	mg/mmol	nmol/mmol	pmol/spot*20 h	nmol/spot*21 h	nmol/spot*21 h	nmol/min*ml
Before HSCT	1	108	236	8049	0.48	1.20	1.44
After HSCT	24	25	72	4788	1.15	1.05	0.66
	38	22	43	6812	0.67	1.23	0.89
	54	27	21	–	–	–	–
Ref. range		8–50	15–30	350–2814	0.02–0.25	0.16–1.41	0.10–0.50

Abbreviations: ASA, Arylsulfatase A; Dpd/crea, urinary deoxypyridinolin-crosslinks/creatinine; GAGs, urinary gylcosaminoglycans; β-Gal, β-Galactosidase*; α*-Idu, *α*-Iduronidase; IDS, Iduronate-2-sulfatase.v

### 2.2 Hematopoietic stem cell transplantation (HSCT)

HSCT was considered given the young age at diagnosis and the parents' strong desire to act on the situation, well-informed on limitations and potential risks. Written informed consent was obtained from both parents. Delayed by a cytomegalovirus (CMV)-infection, the patient underwent HSCT at 9 months of age (Fig. 1B). A male 9/10 human leukocyte antigen (HLA) matched unrelated 37 years old male with an allele mismatch at the HLA-B locus served as bone marrow donor. The graft contained 5.5 × 10^6^ CD34^+^ stem cells per kg recipient body weight and 5 × 10^7^ CD3^+^ T cells per kg recipient body weight. Donor and recipient were matched for blood group (A Rh pos.) and CMV serology. The conditioning regimen consisted of Fludarabin 5 × 30 mg/m^2^ (day −7 until day −3), Thymoglobulin 10 mg/kg (day −6 until day −3), Treosulfan 3 × 14,000 mg/m^2^ (day −6 until day −4), and Thiotepa 2 × 5 mg/kg (day −2). The dose of infused CD34-positive stem cells was 5.5 × 10^6^/kg, the dose of CD3-positive T-cells 50 × 10^6^/ kg. Graft versus host disease (GvHD) prophylaxis was performed with intravenous ciclosporin A and short-course methotrexat.

### 2.3 Follow-up analysis

A valid biomarker to monitor disease progression and therapeutic effects in MLII has not yet been established. Activities of lysosomal enzymes are no effective follow-up parameter considering that the defective GlcNAc-1-phosphotransferase still causes hypersecretion of lysosomal enzymes despite metabolic cross-correction by transplanted donor cells. The lack of lysosomal enzymes in MLII cells results in intracellular accumulation of non-degraded glycosaminoglycans (GAGs), that are also elevated in urine due to lysosomal exocytosis (Table 1). However, urinary GAGs as a storage biomarker can derive from different organs. We therefore additionally analyzed the bone resorption marker deoxypyridinoline (Dpd) as a tissue-specific biomarker. Skeletal alteration in MLII is most likely caused by disturbed bone remodeling, demonstrated by increased levels of urinary Dpd [17]. The amounts of GAGs and Dpd in the patient's urine were measured according to standard procedures and compared to age-matched reference ranges [[14], [18]].

Joint ranges of motion were orthopedically examined at 2.3, 5.3 and 6.3 years of age according to the neutral-zero-method. Plain radiographs were digitally transferred and analyzed using Centricity PACS Universal Viewer (GE Healthcare). Supine anteroposterior pelvic radiographs, performed at birth, 1.3, 2.8 and 3.8 years of age, were evaluated as recently described [3]. Brain and spine magnet resonance imaging (MRI) was done at 1.5, 3.8 and 5.3 years of age.

Formal assessments of neurocognitive development, motor function and adaptive behavior were conducted with Bayley Scales of Infant and Toddler Development®, Third Edition (BSID-III) and Vineland Adaptive Behavior Scales®, Second Edition (VABS-II) at chronological ages of 5.3 and 6.3 years producing age-equivalent scores (AEqs) and developmental quotients (DQ).

## 3 Results

### 3.1 HSCT-related complications and engraftment

The conditioning regimen was well tolerated with anticipated low toxicity. As a result of the transplant volume, the patient experienced a transient transfusion-related acute lung injury. The patient also developed acute GvHD of the mucosa (grade II) and the skin (grade I), which was steroid sensitive and resolved. Myeloid neutrophil and platelet engraftment occurred on days 15 and 31, respectively. The engraftment remained stable with a complete donor-chimerism.

### 3.2 Reduction of pathological urinary GAG and Dpd levels

**Fig. 2:**
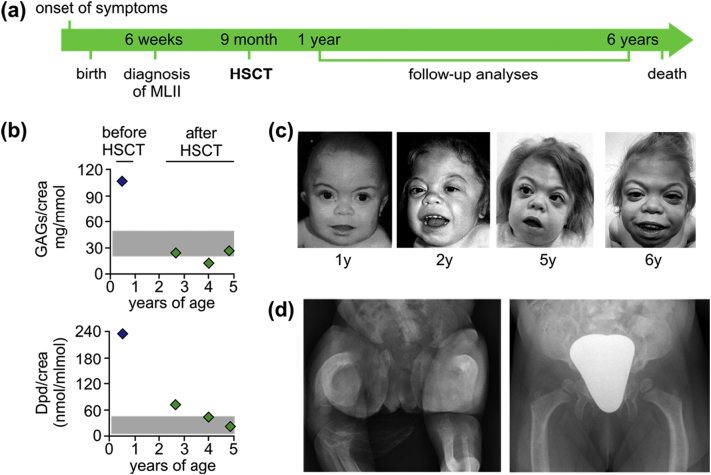
Biochemical and clinical outcome. (**a**) Timeline of the patient history. (**b**) Levels of urinary glycosaminoglycans/creatinine (GAGs/crea) and deoxypyridinoline/creatinine (Dpd/crea) ratios before (blue dots) and after HSCT (green dots). (**c**) Progression of facial disease stigmata. (**d**) Plain pelvic radiography. (left) Radiograph at birth with bowing of the long bones, metaphyseal widening, periosteal cloaking and rickets-like changes of the bones. (right) Radiograph at 2.8 years of age with short acetabular coverage, constriction of the supra-acetabular parts of the os ilium, flared iliac wings, widening of the epiphysis of the proximal femora and mild femoral bowing.

As expected, no relevant HSCT-mediated effect was achieved concerning lysosomal enzyme activities (Table 1). A significant reduction of the GAGs/creatinine levels after HSCT (Fig. 2B, Table 1) indicates that lysosomal enzymes secreted by the donor cells were taken up by defective MLII cells (Fig. 1B). Serial measurements of urinary Dpd/creatinine levels revealed excessive bone resorption before HSCT. After HSCT, these levels normalized (Fig. 2B, Table 1).

### 3.3 Progression of MLII-typical manifestations despite HSCT

Postnatal echocardiography and abdominal ultrasound examinations were unremarkable. Partial respiratory insufficiency was treated with supplemental nocturnal oxygen insufflation. At three years of age, a mucoepidermoid carcinoma was identified in the right submandibular gland, which was surgically excised twice without further treatment. At 6.3 years of age, the patient presented a history of recurrent respiratory infections, but no record of hospitalizations. Cardiac examinations revealed a normal function with trivial mitral and aortic valve thickening and grade I insufficiencies. The patient had developed arterial hypertension and still required nocturnal oxygen and additionally non-invasive ventilation. Abdominal ultrasound showed moderate hepatomegaly and mild renal and splenic calcifications. Ophthalmologic examinations remained unremarkable (i.e. no cornea clouding) apart from alternating esotropia. Facial (Fig. 1D) and overall physical (Fig. S1) disease stigmata had progressed. The patient died at 6.6 years of age following a human metapneumovirus (hMPV) pneumonia and respiratory decompensation on the backdrop of the preexisting partial respiratory insufficiency. Despite early antibiotic, antiviral (ribavirin), immunoglobulin and anti-obstructive (salbutamol, prednisolone, magnesium) therapies, successful fiberoptic intubation and, for the time being, also successful extubation after 30 days of mechanical ventilation, the patient had reached a palliative care situation. Intensive care-related complications involved catheter-associated fungal sepsis, cholestasis and ascites as a result of total parenteral nutrition and tetraplegia presumably due to ischemic brain damage. Eventually, death occurred by muti-organ failure 91 days after hospital admission.

An orthopedic follow-up traced growth cease at 2 years of age. A mild kyphosis was also diagnosed at 2 years and both-sided carpal tunnel syndromes at 5.3 years of age. At age 6.3 years, the patient's weight (12 kg), height (76 cm) and OFC (47 cm) had declined far below the 1st percentile. Correspondingly, the thorax remained very small and narrow. Among the upper extremities, the shoulder joints were affected the most with severe restriction of abduction. The massive outward rotation and limited abduction of the hip joints (passive and active) were the most prominent finding among the lower extremities. Overall, passive ranges of motion stayed stable over the years (Table 2).

**Table 2:** Joint ranges of motion.

	**Passive ranges of motion**		
	at 2.3 y	at 5.3 y	at 6.3 y
Shoulders			
Abduction deficit^1^	40 °	–	20°
IR-AR	–	–	80°-0–30°
Elbows			
Extension deficit^2^	15°	20°	20°
DIP joints D 3–5			
Extension deficit^2^	–	–	30°
Hips			
Extension deficit^2^	25°	–	15°
IR-AR	20°-0–70°	–	20°-0–80°
Abduction	–	30°	30°
Knees			
Extension deficit^2^	5°	left 10°/ right 15°	15°
Ankle joints			
Dorsal extension deficit^2^	–	0°	0°

Abbreviations: D, digitus; DIP joints, distal interphalangeal joints; IR-AR, inward – outward rotation

1 Abduction of 90° was considered as normal.

2 Extension of 0° was considered as normal.

Initially seen pathologies on plain radiography of the hips such as periosteal cloaking, rickets-like changes of the bones and widening of the metaphysis were no longer present at 1.3 years of age. Femoral bowing with coxa vara was prominent at the first assessment and decreased over time. At the last assessment, hip dysplasia with short acetabular coverage and supra-acetabular constriction of the os ilium was seen. Nevertheless, the hips joints (i.e. the containment) stayed stable (Fig. 2D).

**Fig. 3:**
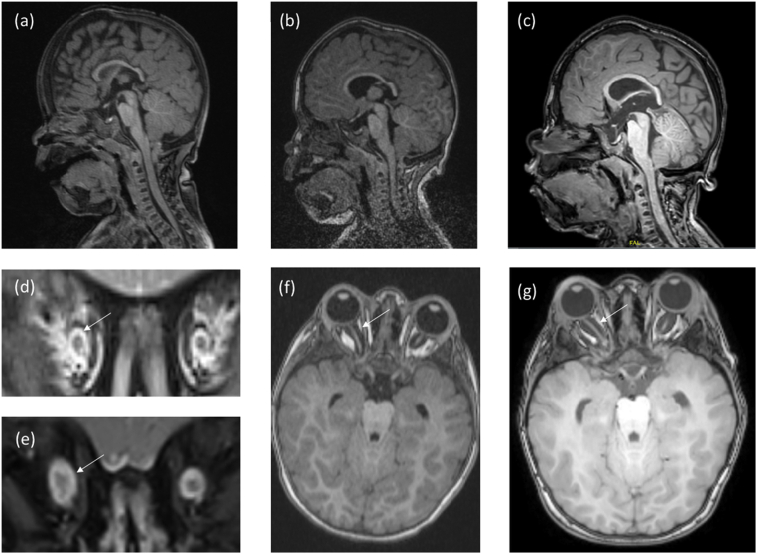
MRI of the brain at 1.5 years (**a**,**d**), 3.8 years (**b**,**f**) and 5.3 years of age (**c**,**e**,**g**). Sagittal T1 images. (**a**,**b**,**c**) show a progressive enlargement of the sella and approximation of the clivus and the pons with increasing flexure of the lower brainstem. The cerebellar tonsils are deep, but do not protrude into the foramen magnum. The subarachnoidal space around the optic nerve is progressively enlarged (Arrows in **d**,**e**,**f**,**g**) without papilledema or signs of increased intracranial pressure. Myelination shows no alteration in T1 (**f**,**g**) or T2 weighted images (data not shown).

MRI performed at 5.3 years of age showed an enlarged and dyplastic sella and a sea horse shaped brain stem with an anteversion of the brainstem and the pons as well as a slight downward displacement of both cerebellar tonsils. No white matter lesions, no enlargement of the perivascular spaces, no brain atrophy, hydrocephalus or progressive cervical spine stenosis were seen. The subarachnoid space around the optic nerves had increased progressively without papilledema (Fig. 3).

### 3.4 Maintained neurocognitive function and quality of life

The patient's overall development progressed continuously since HSCT, albeit very slowly. Independent sitting was achieved by 1.5 years, hand-held walking by 2 years and independent walking by 4.5 years of age. First words were spoken by the age of 2.5 years. At 6.3 years of age, the patient was able to speak bilingually (German, Russian) in short (5-word) sentences and to sing entire songs including lyrics. At chronological ages of 5.3 and 6.3 years, the BSID-III revealed a cognitive function with AEqs of 2.5 (DQ 48%) and 2.3 years (DQ 36%), respectively. Receptive language abilities surpassed expressive ones. Gross motor skills lagged behind. The VABS-II proved low adaptive skills and need of support in most activities of daily living. Nevertheless, the testing reproduced relative strengths in social and communication skills (Table 3). The patient did not complain of any pain and presented as a cheerful child enjoying music, attending kindergarten and engaging in social contacts. No enteral support or invasive ventilation was required.

**Table 3:** Neurocognitive function and adaptive behavior.

		AEqs	
	Domains	at 63 mo	at 75 mo
VABS-II	Communication	24	28
	Daily life skills	27	22
	Social	33	27
	Motor	19	26
BSID-III	Cognitive function	30	27
	Receptive language	32	35
	Expressive language	27	24
	Fine Motor	28	25
	Gross motor	15	17

Abbreviations: AEqs, age-equivalent score; BSID-III, Bayley Scales of Infant and Toddler Development®, Third Edition; DQ, developmental quotient; Adaptive behavior assessed with Vineland Adaptive Behavior Scales®, Second Edition.

## 4 Discussion

This is the first report to specify long-term effects of experimental HSCT in an MLII patient. HSCT might have had an impact on the neurocognitive function and quality of life of our patient. We found no variable effect on growth and skeletal deformities. Short stature and chest deformity still proned for severe cardiorespiratory complications, which are the main cause of death in MLII. Additionally, our patient presented with a mucoepidermoid carcinoma, which would have also significantly reduced life expectancy.

We face several challenges when evaluating the therapeutic effect of HSCT in patients with ultra-rare diseases like MLII. The most important one is the lack of a control group due to very limited natural history data. Several authors reported on progressive psychomotor retardation in MLII [1,9]. However, no longitudinal cognitive data exist in literature and therefore it remains unclear, if MLII is even associated with progressive neurodegeneration. Cathey et al. reported on 14 untreated MLII patients, of whom barely any (1/14) were ambulatory. Verbal expression of almost all (11/12) remained limited to single words, poorly pronounced, and many required enteral support (7/14) and tracheotomy for ventilation (4/14) [2]. In comparison to this, our patient's mobility and quality of life are a rarity as she could walk, communicated well and required no complex medical support besides nocturnal non-invasive ventilation.

After HSCT of our patient, Lund et al. published a retrospective study on 22 transplanted MLII patients indicating that survival may be unchanged since only 27% of the patients had survived six years after HSCT [8]. Three of these patients received bone marrow from siblings with unknown carrier status and two received haploidentical HSCT from a parent. Overall, six of the patients died of organ failure and three of interstitial pneumonia. These data suggest a high risk for transplant-associated complications in this patient cohort. There is little known about optimal conditioning regimens in these children. Some centers favor busulphan, which crosses the blood brain barrier, but comes with some clinically relevant risks, e. g. bronchiolitis organizing pneumonia, vaso-occlusive disease, seizures [19]. Other centers prefer treosulfan-based regimens. While treosulfan itself does not cross the blood-brain barrier, fludarabin and thiotepa are well-known to reach high levels in the brain. The age at HSCT is also considered to be a crucial predictor for success in progressive disorders; it determines the amount of tissue already damaged by the time of transplantation. In MPSIH, earlier HSCT is associated with notably better cognitive outcome [20]. Considering that the diagnosis of our patient was already confirmed by six weeks of age, HSCT at nine months was immensely delayed by the CMV-infection. Taken together, the age at transplantation may impact the outcome. After all, we also did not observe any clear benefit on survival, which seems to remain low after HSCT. The age and cause of death of our patient fits with the natural history [2].

Previously published case reports indicate preservation of cognitive function in follow-ups after bone marrow or cord blood transplantation [9,10]. For example, an MLII patient tested with the Mullen Scales of Early Learning scored cognitive AEqs of 2.0 and 2.6 at chronological ages of 4.7 and 5.7 months, respectively. Taking into account the different time points of testing and the use of different scales, the results roughly equal up with the results from our patient. Unfortunately, this report only covers two time points of neurocognitive testing with no results present from before HSCT. This allows an actual estimate of the patient's global development, but no analysis of any long-term developmental slope. It is noteworthy that poor results in the domains gross and fine motor skills as well as low adaptive skills may also be attributed to physical limitations such as joint contractures. The decision to carry out HSCT in our patient was based on the intention to preserve neurocognitive function. However, due to the lack of natural history data, the extent of cognitive impairment in MLII is unknown and the HSCT-mediated effect cannot be evaluated.

The biochemical examinations reflect an improvement of cellular function. Urinary GAGs may not necessarily be elevated in MLII and a decline over age is physiological even without HSCT [21]. However, growth-independent bone metabolism markers also normalized, putting emphasis on, at least, a previously described tendency of biochemical improvement after HSCT [[9], [10], [11],^13^].

## 5 Conclusions

HSCT might have had an impact on cellular function and MLII disease progression. Five years after HSCT, the MLII patient presented with sustained cognitive function and good quality of life. However, the natural history of MLII is sparsely documented in current literature and formal neurocognitive data lack altogether. We therefore cannot conclude if the clinical outcome of our patient is a consequence of treatment or within the variability spectrum of the phenotype. In order to evaluate experimental therapies, substantial prospective studies and registries of untreated patients are indispensable. Such studies should also include formal neurocognitive testing and neuroimaging.

## Informed consent

Informed consent was obtained from the legal guardians of the patient. They agreed on the use of clinical data and pictures for scientific research and publication.

## Funding

This research received no external funding.

## Declaration of Competing Interest

The authors declare no conflict of interest.

## Appendix A Supplementary data

Supplementary material Image 1
