# Supplementary material for: Is hematopoietic stem cell transplantation a therapeutic option for mucolipidosis type II?
Source: Mol Genet Metab Rep. 2021 Jan 14;26:100704. doi: 10.1016/j.ymgmr.2020.100704 (PMC7815485; doi:10.1016/j.ymgmr.2020.100704)
Supplement: Supplementary file 1 — Supplementary material [file mmc1.docx]

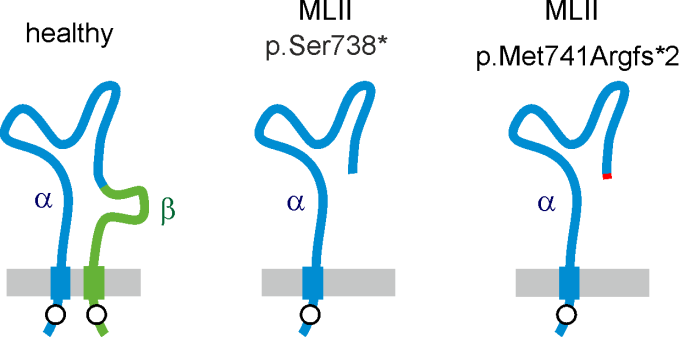


**Fig. S1** Loss-of function mutations in the *GNPTAB* gene of the patient. Both truncated mutant proteins (p.Ser738* and p.Met741Argfs*2) lack the whole β-subunit including the C-terminal transport motif and stay as inactive forms in the endoplasmic reticulum.


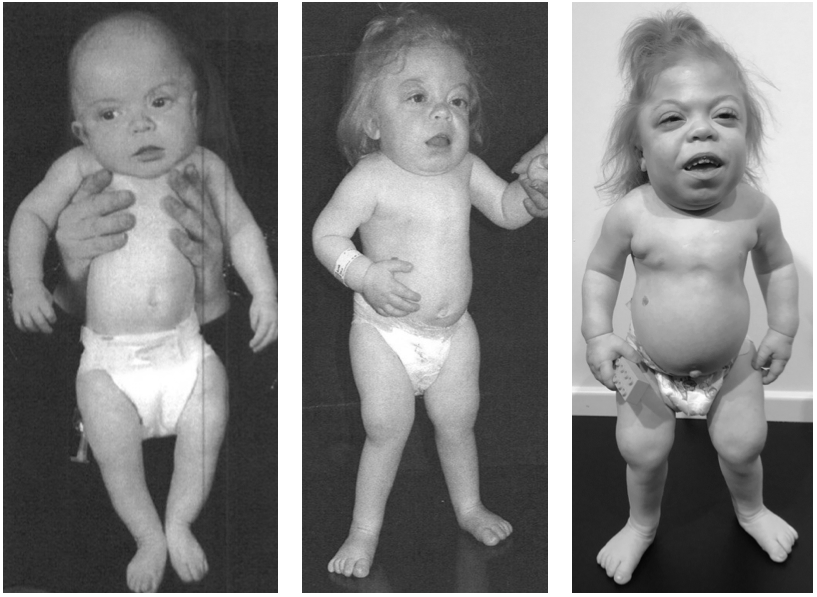


**Fig. S2** General appearance of the patient at 0.3, 2 and 5 years of age.
